# Predicting Abundances of *Aedes mcintoshi*, a primary Rift Valley fever virus mosquito vector

**DOI:** 10.1371/journal.pone.0226617

**Published:** 2019-12-17

**Authors:** Lindsay P. Campbell, Daniel C. Reuman, Joel Lutomiah, A. Townsend Peterson, Kenneth J. Linthicum, Seth C. Britch, Assaf Anyamba, Rosemary Sang

**Affiliations:** 1 Florida Medical Entomology Laboratory, IFAS, University of Florida, Vero Beach, Florida, United States of America; 2 Department of Entomology and Nematology, IFAS, University of Florida, Gainesville, Florida, United States of America; 3 Department of Ecology and Evolutionary Biology, University of Kansas, Lawrence, Kansas, United States of America; 4 Kansas Biological Survey, University of Kansas, Lawrence, Kansas, United States of America; 5 Laboratory of Populations, Rockefeller University, New York, New York, United States of America; 6 Kenya Medical Research Institute, Nairobi, Kenya; 7 United States Army Medical Research Directorate – Africa, Nairobi, Kenya; 8 Biodiversity Institute, University of Kansas, Lawrence, Kansas, United States of America; 9 United States Department of Agriculture, Agricultural Research Service Center for Medical, Agricultural, and Veterinary Entomology, Gainesville, Florida, United States of America; 10 Universities Space Research Association, Columbia, Maryland, United States of America; 11 NASA Goddard Space Flight Center, Biospheric Sciences Laboratory, Greenbelt, Maryland, United States of America; University of Surrey School of Veterinary Medicine, UNITED KINGDOM

## Abstract

Rift Valley fever virus (RVFV) is a mosquito-borne zoonotic arbovirus with important livestock and human health, and economic consequences across Africa and the Arabian Peninsula. Climate and vegetation monitoring guide RVFV forecasting models and early warning systems; however, these approaches make monthly predictions and a need exists to predict primary vector abundances at finer temporal scales. In Kenya, an important primary RVFV vector is the mosquito *Aedes mcintoshi*. We used a zero-inflated negative binomial regression and multimodel averaging approach with georeferenced *Ae*. *mcintoshi* mosquito counts and remotely sensed climate and topographic variables to predict where and when abundances would be high in Kenya and western Somalia. The data supported a positive effect on abundance of minimum wetness index values within 500 m of a sampling site, cumulative precipitation values 0 to 14 days prior to sampling, and elevated land surface temperature values ~3 weeks prior to sampling. The probability of structural zero counts of mosquitoes increased as percentage clay in the soil decreased. Weekly retrospective predictions for unsampled locations across the study area between 1 September and 25 January from 2002 to 2016 predicted high abundances prior to RVFV outbreaks in multiple foci during the 2006–2007 epizootic, except for two districts in Kenya. Additionally, model predictions supported the possibility of high *Ae*. *mcintoshi* abundances in Somalia, independent of Kenya. Model-predicted abundances were low during the 2015–2016 period when documented outbreaks did not occur, although several surveillance systems issued warnings. Model predictions prior to the 2018 RVFV outbreak indicated elevated abundances in Wajir County, Kenya, along the border with Somalia, but RVFV activity occurred west of the focus of predicted high *Ae*. *mcintoshi* abundances.

## Introduction

Rift Valley fever virus (RVFV) is a mosquito-borne zoonotic disease of great economic, livestock, and human health importance in Africa and the Arabian Peninsula [1, 2]. Transmission of RVFV to humans generally involves direct contact with infected tissues or body fluids of animals or bites of infected mosquitoes [3]. Human illness from RVFV often goes unnoticed, or results in flu-like symptoms, but a more severe form of the virus may present, resulting in ocular disease, meningoencephalitis, or hemorrhagic fever, the latter with a case-fatality rate of 50% [4]. Livestock infected with RVFV are less likely to be asymptomatic. High numbers of simultaneous, spontaneous abortions among ruminants (so-called “abortion storms”) and high mortality rates among young animals accompany epizootics [5]. Effects of epizootics on domestic livestock herds are devastating and result in tremendous economic losses due to imposed quarantines and embargo, and food insecurity for communities whose livelihoods depend on livestock [6]. RVFV infection based on serology has also been detected in a wide variety of wild ruminants, from African buffalo to giraffes, but without the pronounced symptoms displayed in livestock [7].

Current RVFV forecasting models use persistence of above-average rainfall, positive Normalized Difference Vegetation Index anomalies, elevated cloud coverage measurements, and the occurrence of El Niño conditions to guide early warning systems [8–13]. Although this approach has seen some success in predicting RVFV risk to general locations and time periods [8, 9, 14–18], model outputs are updated at monthly time steps and do not identify the specific timing or abundances of primary vectors, and thus, potential virus emergence. RVFV epizootics in Kenya are associated with flooding events that result in the emergence of high abundances of *Aedes* mosquitoes, which are considered virus reservoirs [3, 16, 19, 20]. Identifying drivers of high abundances of primary vector species is a logical step toward a better understanding of RVFV ecology, while improving current prediction methods. While high abundances of primary vectors are not the only driver of an RVFV epizootic, information gained from monitoring this component, used in conjunction with existing surveillance efforts, has the potential to contribute to more precise response times when monitoring risk.

Mosquito vectors in the genus *Aedes* are responsible for RVFV maintenance (i.e., primary vectors), while those in the *Culex*, *Mansonia*, and *Anopheles* genera are responsible for RVFV amplification (secondary vectors) in natural environments [21, 22]. Primary vector ecology is a key component in the RVFV disease system, warranting special consideration when inferring risk. Evidence suggests that adult *Aedes* mosquitoes in the subgenera *Neomelaniconion* and *Aedimorphus* transmit the virus transovarially to their eggs, strongly implicating these species as primary disease vectors [3, 19]. Adult *Aedes* primary vector mosquitoes can emerge already infected with the virus and become infectious before feeding on wild or domestic ungulates, thereby establishing low levels of virus activity within a geographic area. If suitable environmental conditions persist, *Culex*, *Mansonia*, and *Anopheles* secondary vector mosquitoes emerge and amplify the virus broadly across vulnerable populations [21]. In Kenya, where 11 epizootics occurred between 1951 and 2007 [23], the mosquito species *Ae*. *mcintoshi* (included within the subgenus *Neomelaniconion*) has been implicated as a major RVFV primary vector, and it is the only species to demonstrate the capacity for transovarial transmission [19, 24, 25]. These factors, combined with high RVFV prevalence in the species during the 2006–2007 epizootic warrant special focus on *Ae*. *mcintoshi* to investigate primary vector abundances and RVFV ecology in this region [3].

Early field studies provided fundamental information regarding *Ae*. *mcintoshi* population biology and ecology. *Ae*. *mcintoshi* prefer *dambo* habitats: shallow depressions in the landscape that become flooded following heavy rainfall [26, 27]. Linthicum *et al*. [28] flooded a dambo artificially for 18 continuous days and found that female emergence occurred at ~14 days, and blood feeding at ~18 days; mosquito life expectancy was <45 days, female dispersal was low (~0.15 km), and direction of dispersal did not always correspond to surface winds. Logan *et al*. [29] conducted a sequence of artificial flooding events and found that ~90% of *Ae*. *mcintoshi* eggs hatched during the initial flooding of a dambo habitat, with fewer eggs hatching in subsequent flooding periods. Our study uses the biological knowledge gained from these initial field studies to predict *Ae*. *mcintoshi* adult abundances across broader landscapes and at different time periods, using georeferenced mosquito abundance data, remotely sensed environmental variables, and a predictive modelling approach.

Specifically, we investigated effects from land surface temperature, cumulative precipitation, compound topographic wetness index values, and percent clay in the soil on adult *Ae*. *mcintoshi* abundances, using zero-inflated negative binomial regression and a multimodel averaging approach. We hypothesized that wetness index values, absolute values of land surface temperatures, and cumulative precipitation would all have positive effects on subsequent adult abundance. High wetness index values indicate greater potential for pooling of water during a precipitation event, a factor important to the initiation of *Ae*. *mcintoshi* hatching and development. Positive correlations between elevated temperatures and mosquito development warranted the inclusion of temperature as a variable in our analyses, and greater cumulative precipitation promotes flooding and standing water. Additionally, we hypothesized that because soils with more clay retain water better, higher percent clay would be associated with lower probabilities of so-called structural zeros in the zero-inflated model framework (see Methods). Having parameterized models with available *Ae*. *mcintoshi* survey data, we used the model to predict *Ae*. *mcintoshi* abundances retrospectively across Kenya and western Somalia. We then observed visual correlations between the timing and locations of high predicted *Ae*. *mcintoshi* abundances and RVFV activity reported in the literature and through public and veterinary health reporting systems, and we compared retrospective predictions across a 16-year time period (2002 to 2018) to observe general differences between predicted values during epizootic and inter-epizootic time periods.

## Methods

Rainfall varies greatly across Kenya, with more moisture in the West. Kenya experiences two rainy seasons referred to as the short rains (October to December) and the long rains (March through May), that coincide with movement of the intertropical convergence zone (ITCZ) [30]. More rainfall occurs from October to December during warm *El Niño* Southern Oscillation (ENSO) anomalies [31].

**Fig 1:**
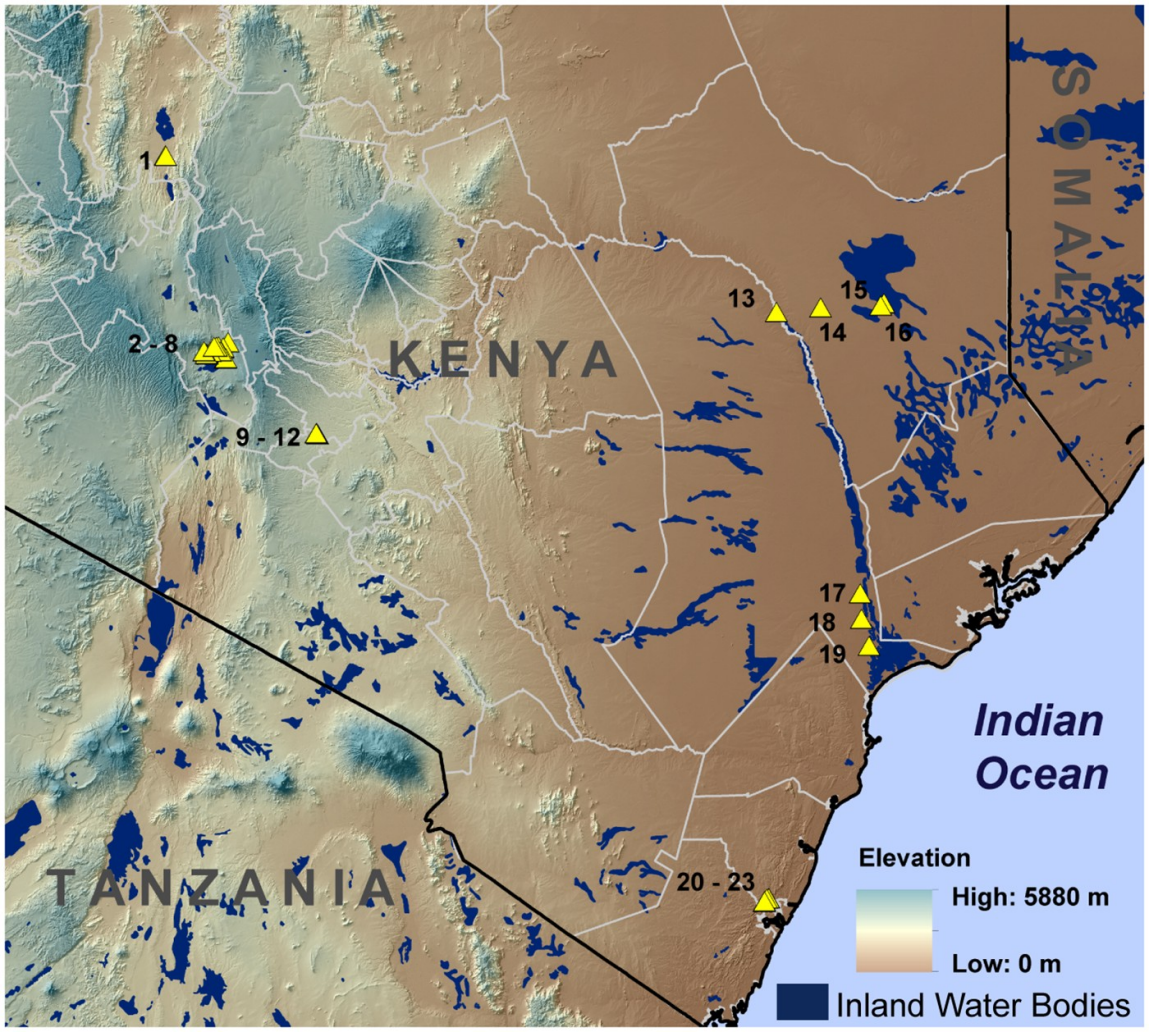
Sample site locations and elevation in study area.

Mosquito abundance data for *Ae*. *mcintoshi* at 23 locations across Kenya were acquired by RS and JL as part of broader ongoing studies in the United States Army Medical Research Detachment in Kenya (Fig 1). Access to locations did not require official permits but was obtained through informal permission from nearby community leaders. Locations selection included areas where arbovirus disease incidence, outbreaks or serosurveillance were reported through previous reports or publications. Insets of sampling locations at a finer scale are available in supplementary materials (S1 Fig). The data consisted of repeat daily sampling at each location for up to 10 sampling days usually twice yearly during the short rains and long rains from 2007 to 2012, except for 2011. One CO_2_-baited CDC light trap was placed overnight at each sample site, and trapped mosquitoes were identified to species by Kenya Medical Research Institute entomology personnel using taxonomic identification keys [32–35].

**Fig 2:**
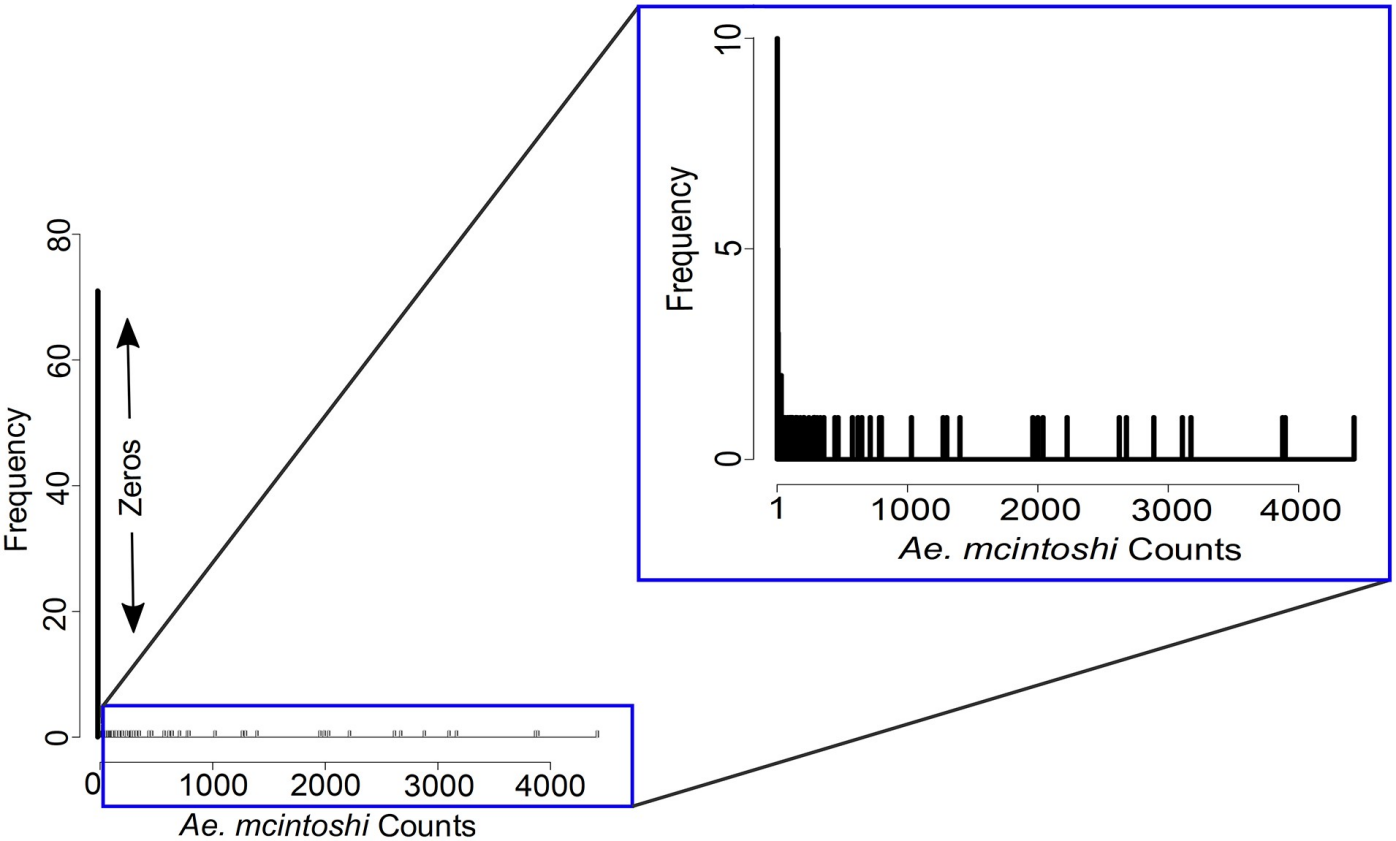
Count frequencies showing frequency of zeros; inset count frequencies from 1 to 4,426.

A total of 158 sampling days were accumulated across all locations during the study period, with abundance values ranging from 0 to 4,426 individuals; dates on which sampling took place but the species was not recorded were assigned an abundance value of zero; such zero counts represented 41% of the total counts (Fig 2).

We obtained Land Surface Temperature/Emissivity data from the Moderate Resolution Imaging Spectroradiometry (MODIS) sensor onboard the Aqua satellite at an 8-day temporal resolution and 1 km spatial resolution [36] through the Reverb ECHO NASA data portal (http://reverb.echo.nasa.gov/reverb). Climate Hazards Group Infrared Precipitation with Stations (CHIRPS) data were obtained through the University of California at Santa Barbara data portal at a daily temporal resolution and a 5 km spatial resolution (http://chg.ucsb.edu/index.html). Compound topographic wetness index values [37] were derived from Shuttle Radar Topography Mission (SRTM) version 4.0 data at a 90 m spatial resolution accessed through the Consultative Group on International Agricultural Research Consortium for Spatial Information (http://srtm.csi.cgiar.org/). Higher wetness index values occur in areas with low slopes and high flow accumulation, which are indicative of locations where water could pool during a precipitation event. Minimum and maximum wetness index values within a 500 m radius of each sampling location were calculated. Percentage of soil clay content was obtained at a 1 km spatial resolution from the Global Soil Dataset for Earth System Modeling Soils [38].

Environmental values at the geographic location of each sample site were extracted from associated raster layers using the raster package in R (Table 1) [39]. Daily cumulative precipitation data corresponding to sampling dates were constructed in three time windows: sampling date to 14 days prior, 14 to 18 days before the sampling date, and 14 to 28 days prior. Land surface temperature data were acquired in 8-day mean composites. We identified the date of sampling and then subtracted 8, 16, and 24 days from the sampling date. The composite data with dates closest to this subtracted value were used in the analysis (Table 1).

**Table 1:** Environmental variables included in candidate models.

Variable	Spatial Resolution	Temporal Period
Minimum Wetness Index	90 m aggregated to 500 m	Static
Maximum Wetness Index	90 m aggregated to 500 m	Static
8 day Land Surface Temperature	1 km	8 day
16 day Land Surface Temperature	1 km	8 day
24 day Land Surface Temperature	1 km	8 day
Cumulative Precipitation 0–14	5 km	Daily
Cumulative Precipitation 14–18	5 km	Daily
Cumulative Precipitation 14–28	5 km	Daily
Percent Clay in the Soil	1 km	Static

Our count data included a greater number of zeros than may be expected under a Poisson or negative binomial distribution (Fig 2). Ignoring this phenomenon would have led to large biases in estimated parameters and their standard errors, and zeros can contribute to overdispersion [40, 41], so we used a zero-inflated statistical modelling approach. Zeros may result from inevitable ecological factors or human error, including sampling error, observer error, or situations in which suitable habitat is present, but is not occupied due to essentially random events [41]. Following standard zero-inflated modelling approaches, we refer to zeros recorded owing to inevitable circumstances as structural zeros, and to those recorded by chance due to sampling variation as sampling zeros [40]. The zero-inflated regression models that we used are mixture models that fit processes for both structural and sampling zeros [42]. Yeşilova et al. [43] described zero-inflated negative binomial regression using

$$\text{Pr}\left(z_{i}|x_{i},y_{i},\alpha ,\beta ,\gamma \right)=\left\{\begin{array}{ll} \pi_{i}+\left(1-\pi_{i}\right){\left(1+\alpha \mu_{i}\right)}^{-\alpha^{-1}}, & z_{i}=0\\ \left(1-\pi_{i}\right)\frac{\Gamma \left(z_{i}+\frac{1}{\alpha }\right)}{z_{i}!\Gamma \left(\frac{1}{\alpha }\right)}\frac{{\left(\alpha \mu_{i}\right)}^{z_{i}}}{{\left(1+\alpha \mu_{i}\right)}^{z_{i}+\frac{1}{\alpha }}}, & z_{i}>0 \end{array}\right\}$$

where *z*_*i*_ is the dependent variable (*Ae*. *mcintoshi* counts at location and sampling day *i*), *π*_*i*_ represents the probability of structural zeroes, *μ*_*i*_ is the expected count at location and sampling day *i* if a structural zero does not occur, and *α* is the overdispersion parameter (*α* ≥ 0). *π*_*i*_ and *μ*_*i*_ depend on the covariates, here denoted *x*_*i*_ and *y*_*i*_, in this way: *μ*_*i*_ = exp(*β*_0_ +*β*_1_*x*_1*i*_ + ⋯ + *β*_*k*_*x*_*ki*_) and *π*_*i*_ = *λ*_*i*_/(1+ *λ*_*i*_) for *λ*_*i*_ = exp(*γ*_0_ +*γ*_1_*y*_1*i*_ + ⋯ + *γ*_*l*_*y*_*li*_) for parameters *β* and *γ*. Zero-inflated regression modelling is a standard technique used in ecology and entomology [43–45]; implementations are available in several software packages.

We evaluated the importance of relationships between mosquito abundances and our various environmental variables using an Akaike’s Information Criterion (AIC) and a Bayesian Information Criterion (BIC) approach [46–48]. Lower AIC or BIC scores indicate better-supported models. AIC model weights (denoted AIC_w_) were also calculated; these values sum to 1 across all models and indicate the weight of evidence supporting a model. Likewise, BIC weights (BIC_w_) were computed. Best-performing models were considered the so-called 99% confidence set of models [47], i.e., for AIC, the models with highest AIC_w_ values and with a cumulative sum of these weights just exceeding 0.99. Importance of predictors was assessed using a standard sum-of-weights approach [47]: the sum of AIC_w_ (respectively, BIC_w_) values for all models in the 99% confidence set that contained a given predictor was computed, this sum being an index of importance of that predictor. Model-averaged coefficients were computed using a weighted average of the coefficients of the models in the 99% confidence set, with weights the AIC_w_ (respectively, BIC_w_) values. We fit zero-inflated negative binomial models to a random sample of 80% of our data set (n = 127), assigning *Ae*. *mcintoshi* abundance values as the response variable and the assembled environmental data as predictor variables (with different subsets of variables used in different models). All models were fitted using the zeroinfl function specifying a negative binomial distribution with a logit link in the pscl package in R 3.13 [49].

We calculated a Pearson’s correlation matrix to assess the potential for multicollinearity between environmental variables, and found high correlation values within, but not between, sets of variables (S1 Table). Only one land surface temperature variable, precipitation variable, or wetness index variable was included in a candidate model at the same time, and we included percent clay in the zero-inflated portion of the model to investigate our hypothesis that zeros recorded in areas where water is likely to pool have a higher probability of being “sampling” zeros, while zeros recorded in areas where water is not likely to pool will have a greater probability of being a “structural” zero.

We investigated model residuals from the lowest-AIC model for evidence of spatial autocorrelation using a spline correlogram in the ncf package in R [50, 51], and found no evidence of it (S2 Fig). Although sampling dates were inconsistent across study site locations and time periods, we investigated the potential for residual temporal autocorrelation, but did not find obvious temporal patterns in residuals.

Estimated parameters from best performing models were used to predict mosquito abundances using a random sample of 20% of the abundance data (n = 31) withheld from the regression models, and a root mean square predictive error was calculated to evaluate accuracy. We used the predict function in the pscl package, with type = “response” to incorporate both the structural zero portion and the sampling portion of the zero-inflated negative binomial model when predicting values [49, 52].

For retrospective predictions, unsampled locations were generated across the study region at 10 km intervals, predictions were generated from each model in the 99% confidence set, and a weighted average of predictions was computed using the AIC_w_ values as weights. Predicted values were rasterized into a 10 x 10 km grid for visualization purposes. Values greater than 50,000 were considered extrapolative and excluded from visualizations, based on the fact that the greatest number of mosquitoes trapped in a single day across all species in our study locations was 47,694. Values > 50,000 constituted a very small percentage (~0.01%) of predicted values in each raster and occurred only at the fringes and across portions of large water bodies, presumably, where extreme compound topographic wetness index values are present. Data and sample R code are available in S1 Data Code.

## Results and discussion

Model results corroborated several factors known from local-scale studies to be important for *Ae*. *mcintoshi* development and population ecology. The 99% confidence set of models with respect to AIC consisted of 11 models (Table 2; full model results in S2 Table), and, with respect to BIC, of 19 models (S2 Table); these are the models best supported by data according to each information criterion, with the support for each model quantified by its AIC (or BIC) weight (AIC_w_ or BIC_w_). The same variables tended to be included as predictors in the best-supported models according to both AIC and BIC, and model-averaged coefficients were similar (S3 Table), so the choice of information criterion did not substantially affect results. We henceforth use AIC because our objectives include prediction, for which AIC is considered more suitable [53, 54].

**Table 2:** Model averaged coefficients and sums of AIC_w_ for each candidate variable using the 11 best models. AIC_w_ values close to one indicate strong support for the importance of a predictor.

	Negative Binomial Model Component									Zero-Inflation	
*Variable*	Intercept	Wetness Index Max	Wetness Index Min	Precip 0 to 14 days	Precip 14 to 18 days	Precip 14 to 28 days	Land Surface Temp 8	Land Surface Temp 16	Land Surface Temp 24	Intercept	% Clay
*Model averaged coefficient*	-11.858	0.000	1.825	0.010	0.000	0.000	0.003	0.004	0.060	1.202	-0.051
*Sum of AIC*_*w*_	0.994	0.000	0.994	0.870	0.004	0.042	0.061	0.072	0.819	0.994	0.994

The sum of AIC_w_ across the 11 best models including a given predictor indicated the importance of that predictor; these sums showed that minimum wetness index values within 500 m of a sampling site, cumulative precipitation from the sampling date to 14 days prior, land surface temperature values from 8-day mean composites 24 days prior to sampling, and percentage of clay in the soil (in the structural-zero portion of the model—see Methods) had the greatest importance for predicting abundance (Table 2).

The data strongly supported a positive relationship between minimum wetness index values within 500 m of a sampling site and mosquito abundance (Table 2). This result corroborated existing information regarding *Ae*. *mcintoshi* ecology: locations with high minimum wetness index values have low slopes and high flow accumulation, indicative of *dambo* habitats or landscapes likely to collect water during a precipitation event. We also found a positive effect on mosquito abundance of cumulative precipitation from the sampling date to 14 days prior, and no meaningful effect of cumulative precipitation 14 to 18 or 14 to 28 days prior to sampling. These results indicated that precipitation within a short time period prior to emergence has a greater impact on *Ae*. *mcintoshi* abundances, suggesting that forecasting may be enhanced with the addition of shorter time intervals. Model results indicated a positive effect on mosquito abundances of 8-day mean land surface temperature composites at 8, 16, and 24 days prior to sampling, with 24 days prior having the greatest effect among these variables. Temperature is known to play an important role in mosquito life stage development and may impact RVFV vector competency in some species [55, 56]. Our study results suggest that warmer land surface temperatures a few weeks prior may produce conditions that subsequently contribute to higher adult abundances, if precipitation events initiate mosquito development. Interestingly, Anyamba et al. and Linthicum et al. [57, 58] have found negative associations between anomalously lower temperatures and RVFV activity in eastern and southern Africa, suggesting a more complex association between this variable and events leading to epizootic events. We found a negative relationship between percent clay in the soil and the probability of a structural zero within the zero-inflated portion of the model, indicating that areas with low clay content had a higher probability of a structural zero, presumably because standing-water pools are less likely to form over such soil types.

**Fig 3:**
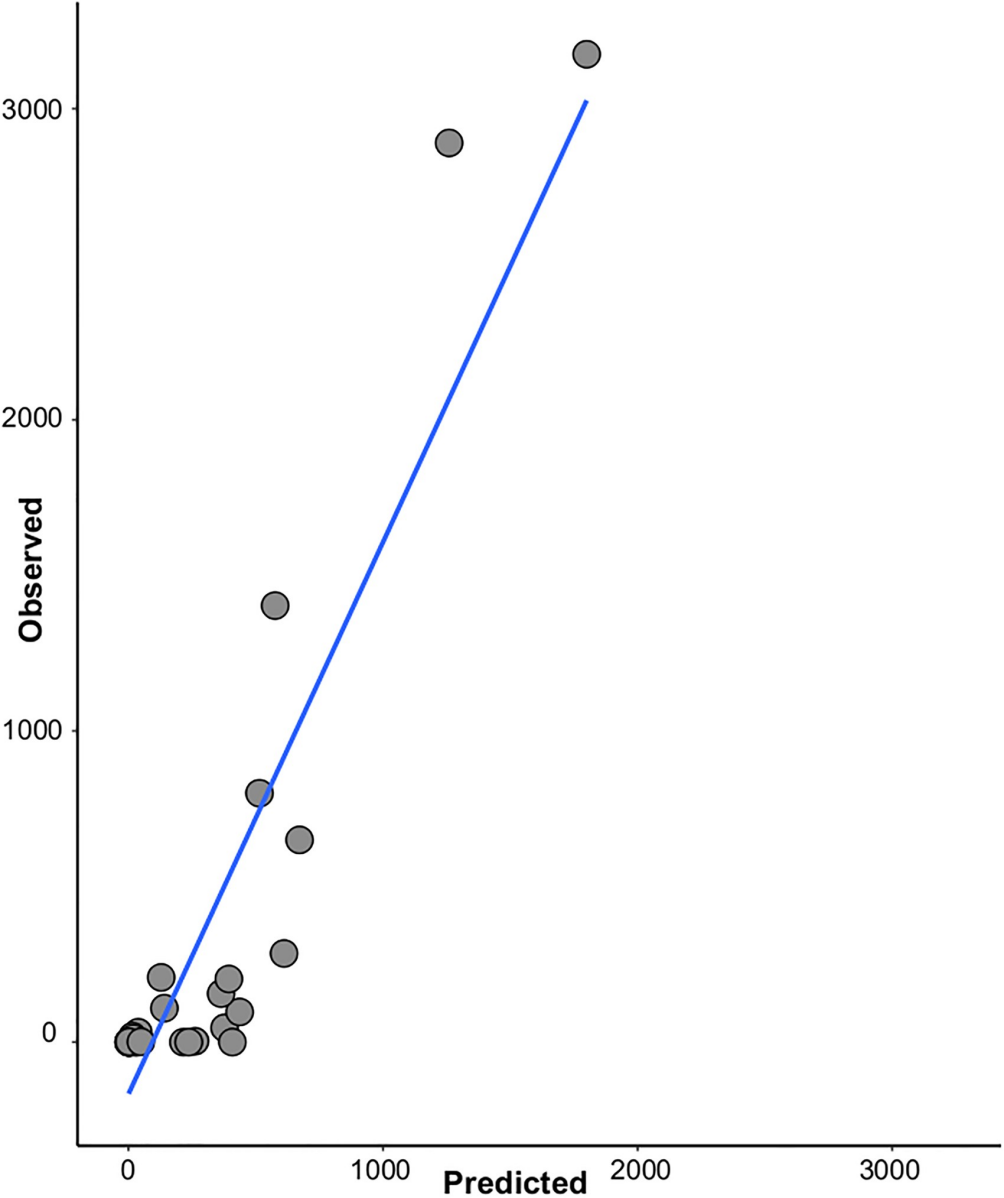
Observed vs. predicted values (n = 31). These are out-of-sample predictions (see Methods).

**Fig 4:**
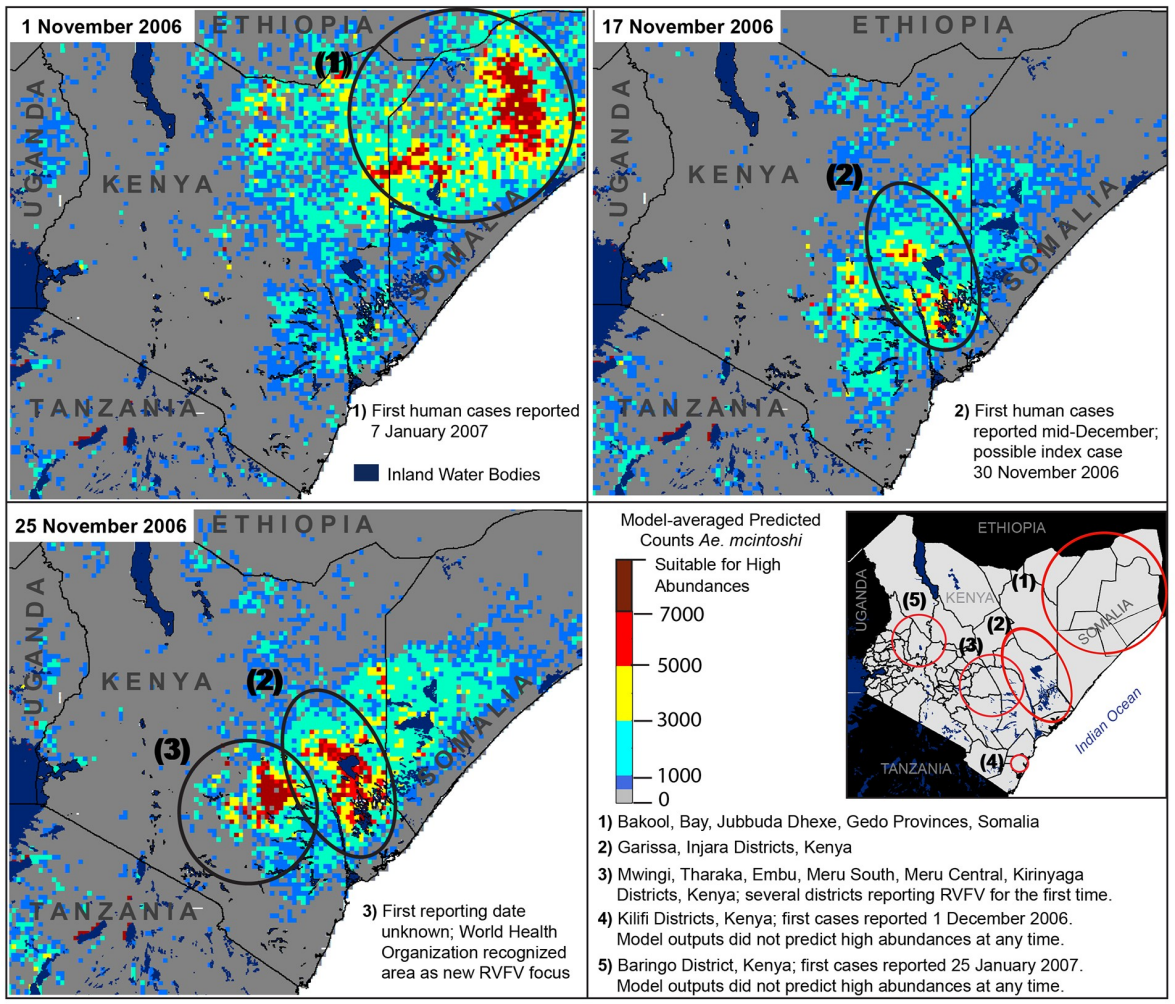
Predicted elevated abundances prior to and during the 2006–2007 epizootic (heat maps) and locations of independently documented RVFV foci (numbered circles). District boundaries fall inside circles in the lower, right panel, with corresponding information provided below the panel map.

Model-averaged out-of-sample predictions demonstrated the capacity of our models to predict elevated abundances (Fig 3). Our models’ predictive accuracy was low for low observed abundances: the relationship between observed and predicted values was weak on the left-most side of Fig 4. However, as the intended use of the models is to predict very high abundances, this point is of secondary importance. Prediction of high abundances was effective, although the model underestimated the observations. Model performance statistics, including root mean square error values from out-of-sample predictions for the 11 best models are provided in more detail in S2 Table.

Retrospective model-averaged vector abundance predictions were produced at 8-day intervals from 30 September 2002 through 25 January 2018, and model predictions were generated from 25 January 2018 to August 2018 because of anomalous precipitation events, resulting in a RVFV epizootic during this time period. Monitoring and human health agencies such as the World Health Organization reported [59–61] high abundances of RVFV cases at five times and locations during the 2006–2007 epizootic period (Fig 4, numbered circles). Retrospective vector abundance predictions were high pre-dating three of these, while predictions were low prior to two RVFV foci (Fig 4, heat maps). Elevated model predictions prior to the 2018 RVFV epizootic suggested two time periods in the month of April in which *Ae*. *mcintoshi* abundances may have been elevated, but the locations of theses predicted abundances were approximately 125 km from the first RVFV focus. Additionally, very high abundances were predicted along the southeastern coast of Kenya, where outbreaks were not reported. These results highlight the complexity of RVFV transmission in space and time and warrant additional investigation into the role that primary vector activity as a precursor to epizootic events, especially when immune status of animals and /or their vaccination history preclude viremia and a disease outbreak in these animals. Notable model outputs during epizootic and inter-epizootics are described in more detail, and a timeline between predicted abundances and epizootic activity is provided in supplementary materials (S4 Table).

Elevated abundances were predicted for 1 November 2006 in Bay, Bakool, Jubbuda Dhexe, and Gedo Provinces, Somalia (Fig 4, circle 1), ~7 weeks prior to reports of human cases [62]. Our results support virus circulation within Somalia independent of Kenya, with high vector abundance predictions in isolated areas prior to elevated predicted abundances in Kenya (Fig 4). Although, the ecological habitats and some of the drainage systems in Kenya and Somalia were essentially identical it is possible that flooding of mosquito habitat in Somalia occurred before habitats in Kenya due to rainfall events. High abundances were predicted for 17 and 25 November in Garissa, Ijara, and Kitui Districts, Kenya; human cases of RVFV were first reported in mid-December in Garissa, with the potential index case presenting on 30 November (Fig 4, circle 2) [63]. Additionally, several of the Kenyan districts located within high predicted abundance areas for 25 November later reported RVFV activity for the first time: Kitui, Tharaka, Mwingi, Embu, Kirinyaga, Meru South, Meru Central, and Malindi District (Fig 4, circle 3) [60]. The World Health Organization recognized cases in these districts as a new RVFV focus during the outbreak, although little information is available regarding the exact date of onset of symptoms in humans or animals [61]. It is important to note that the Food and Agricultural Organization of the United Nations published a RVFV outbreak alert in October 2016 (see [64] and www.geis.fhp.osd.mil/GEIS/SurveillanceActivities/RVFWeb/indexRVF.asp) for East Africa based upon the NASA/USDA/DoD RVFV risk prediction model (https://ars.usda.gov/saa/cmave/rvf) indicating elevated risk in September of 2016 [15].

Human RVFV cases were first reported retrospectively on 6 December 2006 in Kilifi District, near the eastern coast of Kenya (Fig 4, circle 4) [63]. Our models did not predict high *Ae*. *mcintoshi* abundances at any time in this area. Nguku et al. (2010) [63] found that illness in Kilifi District coincided with heavy rainfall, rather than emerging approximately one month after heavy rainfall as was the case in other regions, and that movement of infected livestock from the outbreak area in Northeastern Province, Kenya, into Kilifi District may have been the catalyst for the outbreak. These observations suggest that primary vector emergence in Kilifi District may not have been responsible for virus circulation in the area, but further investigation is needed.

Illness in Baringo District was estimated to begin in late December, 2006, with the first human case reported on 25 January 2007 [60, 63], but our model results did not predict elevated *Ae*. *mcintoshi* abundances in Baringo District at any time during the study period, suggesting that other species may have played a role in driving the RVF transmission (Fig 4, circle 5). Although it is possible that our model predictions did not reflect the potential for *Ae*. *mcintoshi* abundance in this region, more likely the model was accurate and suitable habitat for this species was deficient in the area. Low numbers of *Ae*. *mcintoshi* were collected in Baringo District during the 2006–2007 epizootic, and follow-up sampling also retrieved low densities of this species [3, 27]. Our data set had only two records for *Ae*. *mcintoshi* in Baringo District with 2 and 6 females recorded, respectively. Despite the low numbers of *Ae*. *mcintoshi* collected in our sample data set, this result likely had to do with the timing and sampling effort in this area, as *Ae*. *mcintoshi* is known to be present and abundant in this region at various time periods (KJL personal communication). Additional sampling is required to capture environmental conditions that produce high numbers of *Ae*. *mcintoshi* in Baringo District in order to predict abundances accurately.

**Fig 5:**
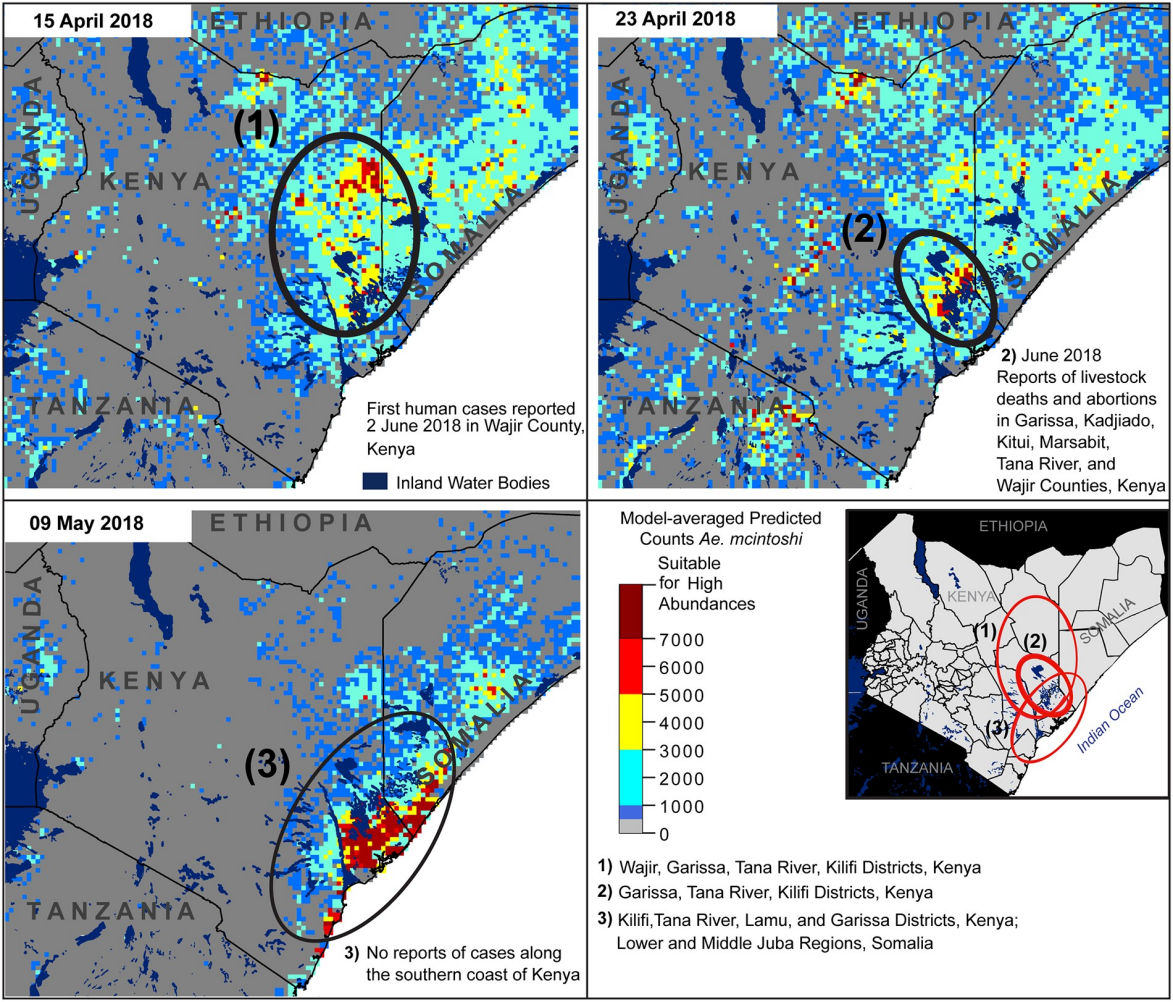
Predicted elevated abundances prior to the 2018 epizootic (heat maps) and locations of independently documented RVFV foci (numbered circles 1,2); areas with high predicted abundances, and no reports of RVFV activity (numbered circle 3). District boundaries fall inside circles in the lower right panel, with corresponding information provided below the panel map.

In February 2018, unusually heavy rainfall activity occurred in Northeastern Kenya; County Directors were put on alert for RVF activity, and a national alert was issued 30 May 2018 [65, 66]. The first human RVF case entered a medical facility in Wajir County on 2 June, and an RVF outbreak was declared on 8 June [65]. Subsequent cases followed in Garissa, Kadjiado, Kitui, Marsabit, and Tana River Counties. Model predictions from February to June 2018 indicated high predicted *Ae*. *mcintoshi* abundances on 15 April in Wajir County on the border with Somalia, approximately 6–7 weeks prior to the RVF outbreak, but the area with highest predicted abundances was ~125 km southeast of the outbreak area (Fig 5). Slightly elevated abundances were predicted for the following week in Garissa and Tana River Counties. Very high predicted abundances on 9 May 2018 spanned southern Garissa and Lamu Counties on the coast, and east into Somalia.

**Fig 6:**
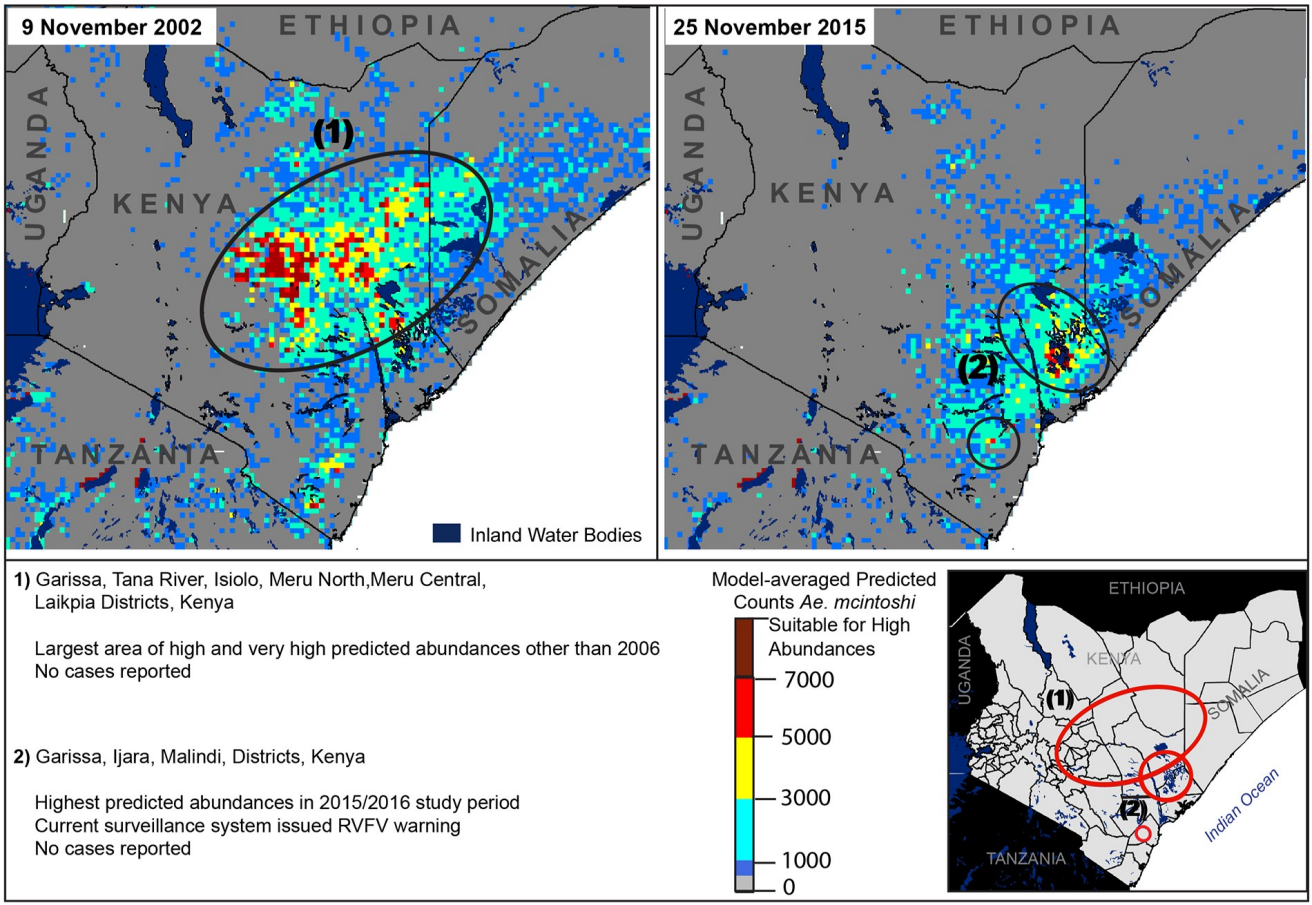
Predicted elevated abundances during interepizootic year 2002 (left panel), and lower predicted abundances during the 2015/2016 time period, when current RVFV surveillance systems issued warnings, but no virus was reported (right panel). District boundaries fall inside circles in the lower right panel, with corresponding information provided below the panel map.

**Fig 7:**
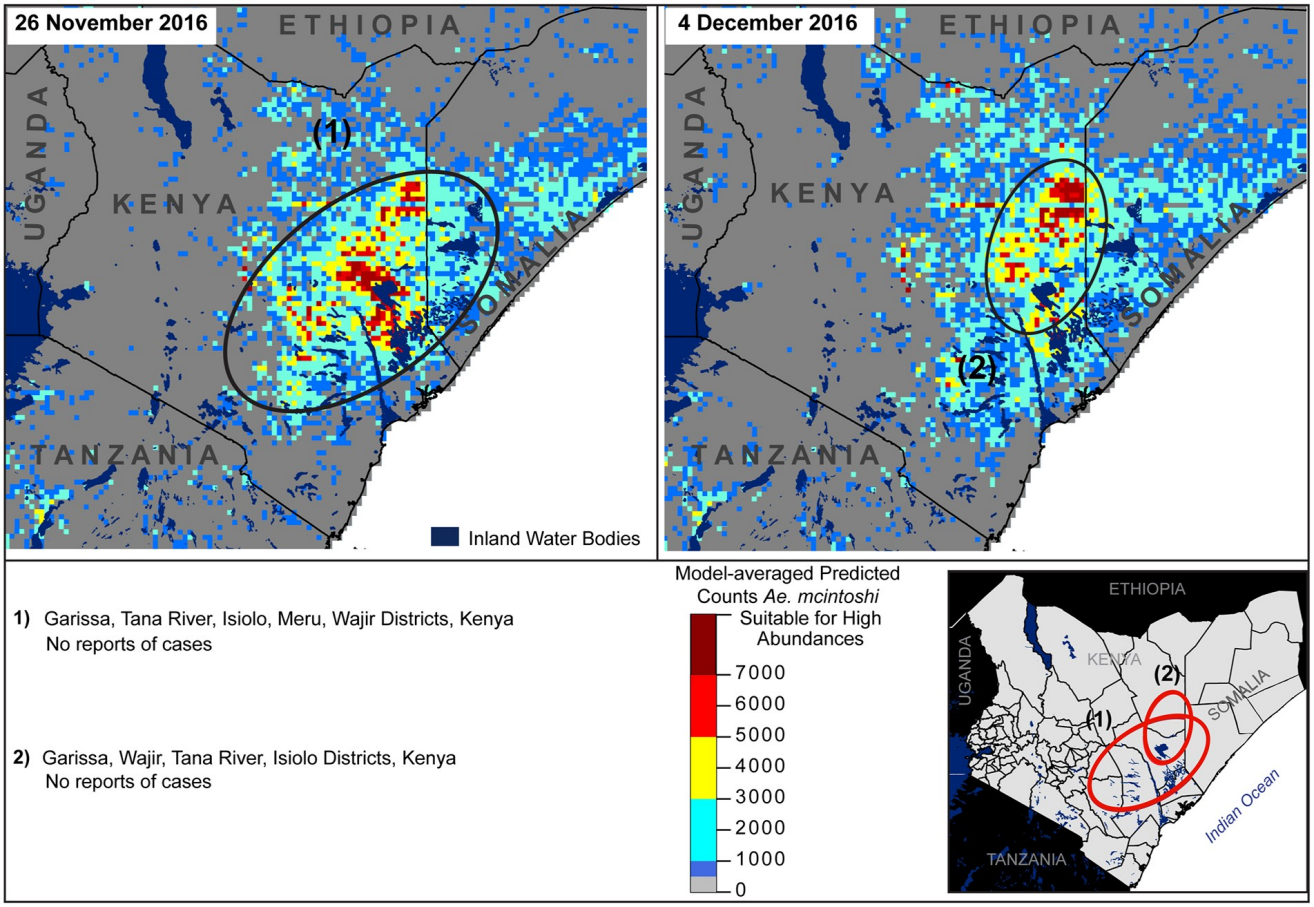
Predicted elevated abundance during late November (left panel) and early December 2016 (right panel); no virus activity reported, widespread vaccination occurred in 2015/2016 short rains season. District boundaries fall inside circles in the lower right panel, with corresponding information provided below the panel map.

**Fig 8:**
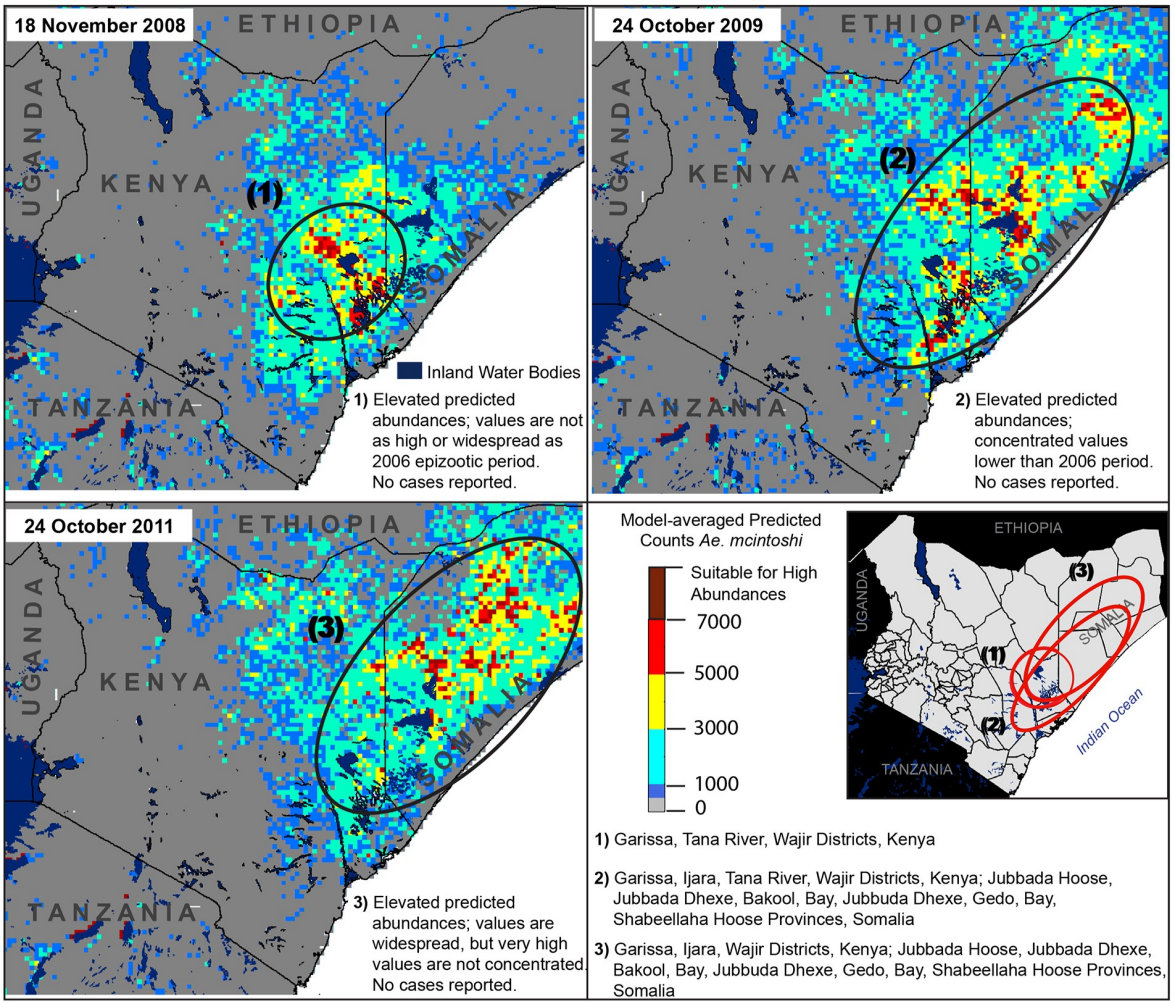
Medium-scale predictions of elevated abundances which apparently did not result in any reported RVFV cases, including 18 November 2008 (upper left), one week after animal health authorities issued an RVFV warning in the far northeastern region of Kenya. District boundaries fall inside circles in the lower right panel, with corresponding information provided below the panel map.

Comparative predictions during the short rain season between 2002 and 25 January 2018 indicated lower predicted abundances across the study period (S1 Movie File), with the exception of one date in 2002 (Fig 6) and two dates in 2016 (Fig 7), and a few small- and medium-scale predictions of elevated abundances which apparently did not result in reported RVFV cases (Fig 8). High predicted abundances from 9 November 2002 were located to the northwest of the 2006–2007 focus in Isiolo and Laikipia Districts, Kenya (Fig 6, circle 1); virus activity was not reported there during this time period, nor was it reported in the regions shown in Figs 7 and 8. Even though conditions appeared to be suitable for high primary vector emergence, the possibility exists that ova deposited in these areas were not infected with RVFV transovarially and did not emerge with the capacity to transmit the disease; conditions were not suitable for large numbers of secondary vectors to amplify the virus; or susceptible livestock were not present in the area. In fact, some evidence indicates possible livestock movement restrictions in this area and during this time period because of rinderpest virus detected in cattle on 23 October 2002 [67], but further investigation is needed before determining whether this factor could have impacted potential RVFV circulation.

Importantly, abundances predicted by our models were low during two time periods in which RVFV warnings were issued in Kenya but no virus activity was reported, demonstrating the contribution of investigating RVFV at multiple spatial and temporal scales. Animal health authorities issued a warning on 13 November 2008 in the far northeastern region near the borders of Ethiopia and Somalia because of persistent rainfall. Our model did not predict high vector abundances in this region prior to this date. The highest abundances predicted around this date were only moderate and were south of this region (Fig 6, circle 1). Several national and international agencies issued warnings for potential RVFV activity in the 2015–2016 period because of an *El Niño* event in the study area [68], but we did not predict high abundances during this time period (Fig 5, right panel) and no outbreaks were reported. Interestingly, very high abundances were predicted for 26 November 2016 and 4 December 2016 (Fig 7), but again no outbreak was reported. One likely possibility is that implementation of widespread livestock vaccination program occurred in the lead up to the 2015 *El Niño* event provided a substantial level of herd immunity throughout 2016 [18]. Integrating model predictions with livestock restocking rates and immunity status into a broader model of RVF potential will provide a more grounded assessment of virus risk than estimates of primary vector abundances alone. Weekly predictions during the short rain season from 1 September to 25 January between 2002 and 2018 are available in the supplementary material (S1 Movie File).

Although our model can predict high mosquito abundances, our data set does not appear to capture the breadth of environmental conditions suitable for *Ae*. *mcintoshi* in central or western regions of Kenya, highlighted in the absence of high predicted values during the 2006–2007 epizootic in Baringo District and the collection of high abundances of this species in the past (Linthicum and Anyamba, personal communication). The very low number of *Ae*. *mcintoshi* collected from this region was likely due to the sampling effort available in the data set used for model calibration, demonstrating the need for additional and regular sampling to capture conditions that contribute to large emergences of *Ae*. *mcintoshi*.

In addition to this limitation, model predictions do not incorporate mosquito density dependence, which may affect the accuracy of predicted values, particularly at higher values. Additionally, we have lower confidence in predicted values greater than ~7,000 because of the potential for model extrapolation. The choice of 7,000 individuals is a user defined threshold and should be interpreted as such. Predicted values greater than the maximum 4,426 individuals in our data set could be interpreted as extrapolation, but we assume that the possibility exists for a greater number of individuals to be collected in a single trap night. This threshold allows for predicted values to exceed the maximum individuals in our data set, while providing a value at which to indicate visually when and where much higher predicted values occur in model outputs. Input data consist of irregular sampling in space and time. This sampling scheme proved challenging to assess the potential for spatial and temporal autocorrelation that may affect parameter estimates, but we did not find evidence of this phenomenon. We predict mosquito abundances across a relatively broad spatial region, which also increases the potential for extrapolation; although, the combination of environments across the prediction area are not unlike those encountered at the sampling sites, and we assume that the effect of these environments on mosquito abundances are similar to the effect of these environments on the sample data, except in the central and western portion of the study area, where we determined insufficient data for accurate model predictions. We also predict across a broad temporal period. The irregular sampling scheme, across a five-year time period helps mitigate temporal extrapolation across the predictive time period.

Predicting abundances from mosquito trap data presents challenges because of the potential for bias between individual traps, trap types, and trap bait [69]. Additionally, mosquito traps collect a fraction of the diversity and relative abundances of a mosquito community on a given night. In the case of this study, consistency in trap type and bait used across sampling sites helped to reduce bias from multiple trap types, but uncertainty remains in exact diversity and abundances of mosquitoes in collected areas. Satellite measurements of environmental variables aggregate values across a landscape to a single spatial resolution, even though the potential exists for local variations. Our data consisted of repeat sampling at locations across Kenya, but sampling was sparse in some areas. Our model predicts abundances for one primary RVFV vector, but additional primary vector species likely exist: several species were identified for further investigation during the 2006–2007 epizootic [3]. RVFV surveillance may benefit from applying our modelling framework to those species.

Our model is the first to use predicted vector abundances at an 8-day temporal resolution as a means of predicting primary RVFV mosquito vector emergence in this region. This approach provides a powerful framework to help inform current RVFV monitoring systems. Additionally, our framework has the potential to reveal new information about primary vector activity during inter-epizootic years that may contribute to a better understanding of RVFV ecology. The potential exists to extend our framework to multiple vectors and disease systems. Our approach also allows exploration of the effects of climate change on *Ae*. *mcintoshi* or other medically important vector species. For improved model performance, we recommend conducting mosquito collections at regular and more frequent temporal intervals and that these collections capture greater spatial and ecological diversity throughout RVFV potential epizootic areas.

## Supporting information

S1 Fig Insets of sampling locations at a finer scale. (TIF)

S2 Fig Spline correlogram of distance between locations and model residuals for the lowest-AIC model. The horizontal black line located at 0.0 on the y-axis represents complete spatial randomness; the red line plots the residual correlation from the model over distance; and the additional black lines show 95% bootstrap confidence intervals around the observed residual correlation.
(TIF)

S1 Data Code Data used in analysis and R code to execute model runs. (ZIP)

S1 Table Pearson’s correlation matrix. (XLSX)

S2 Table Candidate models: AIC and BIC rankings, scores, differences, and weights for all candidate models run in this analysis. (XLSX)

S3 Table Model averaged coefficient values for AIC and BIC models corresponding to a cumulative weighted value of 0.994 (11 models for AIC and 19 models for BIC). (XLSX)

S4 Table Timeline between predicted abundances and epizootic activity. (XLSX)

S1 Movie File Animated plots between 1 September and 25 January from 2002 to 2018. (MP4)

## References

[1] Davies F. The Historical and Recent Impact of Rift Valley Fever in Africa. *Am J Trop Med Hyg*. 2010;83(2):73–4. 20682909 10.4269/ajtmh.2010.83s2a02 PMC2913498

[2] Rich KM, Wanyoike F. An assessment of the regional and national socio-economic impacts of the 2007 Rift Valley fever outbreak in Kenya. *Am J Trop Med Hy*g. 2010;83(2 Suppl):52–7. 10.4269/ajtmh.2010.09-0291 20682906 PMC2913501

[3] Sang R, Kioko E, Lutomiah J, Warigia M, Ochieng C, O’Guinn M, et al Rift Valley fever virus epidemic in Kenya, 2006/2007: The entomologic investigations. *Am J Trop Med Hyg*. 2010;83(2 Suppl):28–37. 10.4269/ajtmh.2010.09-0319 20682903 PMC2913497

[4] World Health Organization. Rift Valley fever. 2010;Fact sheet N°207 (http://www.who.int/mediacentre/factsheets/fs207/en/).

[5] Pepin M, Bouloy M, Bird BH, Kemp A, Paweska J. Rift Valley fever virus (Bunyaviridae: Phlebovirus): An update on pathogenesis, molecular epidemiology, vectors, diagnostics and prevention. *Vet Res*. 2010;41(6). 10.1051/vetres/2010033 PMC2896810 21188836

[6] Chengula AA, Mdegela RH, Kasanga CJ. Socio-economic impact of Rift Valley fever to pastoralists and agro pastoralists in Arusha, Manyara and Morogoro regions in Tanzania. *Springerplu*s. 2013;2. 10.1186/2193-1801-2-549 PMC3825084 24255846

[7] Britch SC, Binepal YS, Ruder MG, Kariithi HM, Linthicum KJ, Anyamba A, et al Rift Valley fever risk map model and seroprevalence in selected wild ungulates and camels from Kenya. *PLoS One*. 2013;8(6):e66626 10.1371/journal.pone.0066626 23840512 PMC3695998

[8] Anyamba A, Linthicum KJ, Mahoney R, Tucker CJ, Kelley PW. Mapping potential risk of Rift Valley fever outbreaks in African savannas using vegetation index time series data. *Photogramm Eng Remote Sensing*. 2002;68(2):137–45.

[9] Anyamba A, Linthicum KJ, Tucker CJ. Climate-disease connections: Rift Valley Fever in Kenya. *Cad Saude Publica*. 2001;17 Suppl:133–40. 11426274 10.1590/s0102-311x2001000700022

[10] Linthicum KJ, Anyamba A, Tucker CJ, Kelley PW, Myers MF, Peters CJ. Climate and satellite indicators to forecast Rift Valley fever epidemics in Kenya. *Science*. 1999;285(5426):397–400. 10.1126/science.285.5426.397 10411500

[11] Linthicum KJ, Bailey CL, Davies FG, Tucker CJ. Detection of Rift-Valley Fever Viral Activity in Kenya by Satellite Remote-Sensing Imagery. *Science*. 1987;235(4796):1656–9. 10.1126/science.3823909 3823909

[12] Linthicum KJ, Bailey CL, Tucker CJ, Angleberger DR, Cannon T, Logan TM, et al Towards Real-Time Prediction of Rift-Valley Fever Epidemics in Africa. *Prev Vet Med*. 1991;11(3–4):325–34.

[13] Linthicum KJ, Bailey CL, Tucker CJ, Mitchell KD, Logan TM, Davies FG, et al Application of Polar-Orbiting, Meteorological Satellite Data to Detect Flooding of Rift-Valley Fever Virus Vector Mosquito Habitats in Kenya. *Med Vet Entomol*. 1990;4(4):433–8. 10.1111/j.1365-2915.1990.tb00462.x 1983457

[14] Anyamba A, Chretien JP, Formenty PBH, Small J, Tucker CJ, Malone JL, et al Rift Valley fever potential, Arabian Peninsula. *Emerg Infect Dis*. 2006;12(3):518–20. 10.3201/eid1203.050973 16710979 PMC3291449

[15] Anyamba A, Chretien JP, Small J, Tucker CJ, Formenty PB, Richardson JH, et al Prediction of a Rift Valley fever outbreak. *Proc Natl Acad Sci* USA. 2009;106(3):955–9. 10.1073/pnas.0806490106 19144928 PMC2626607

[16] Anyamba A, Chretien JP, Small J, Tucker CJ, Linthicum KJ. Developing global climate anomalies suggest potential disease risks for 2006–2007. *Int J Health Geogr*. 2006;5:60 10.1186/1476-072X-5-60 17194307 PMC1779293

[17] Anyamba A, Linthicum KJ, Small J, Britch SC, Pak E, de La Rocque S, et al Prediction, assessment of the Rift Valley fever activity in East and Southern Africa 2006–2008 and possible vector control strategies. *Am J Trop Med Hyg*. 2010;83(2 Suppl):43–51. 10.4269/ajtmh.2010.09-0289 20682905 PMC2913499

[18] Anyamba A, Chretien JP, Britch SC, Soebiyanto RP, Small JL, Jepsen R, et al Global Disease Outbreaks Associated with the 2015–2016 El Niño Event. *Sci Rep*. 2019;9(1):1930 Epub 2019/02/13. 10.1038/s41598-018-38034-z 30760757 PMC6374399

[19] Linthicum K, Davies F, Kairo A, Bailey C. Rift Valley fever virus (Family Bunyaviridae, Genus *Phlebovirus*)–Isolations from Diptera collected during an inter-epizootic period in Kenya. *J Hyg*, *Camb*. 1985;95(1):197–209. 10.1017/s0022172400062434 2862206 PMC2129511

[20] Davies F, Highton R. Possible vectors of Rift-Valley fever in Kenya. *Trans R Soc Trop Med Hyg*. 1980;74(6):815–6. 10.1016/0035-9203(80)90213-8 6111141

[21] Bicout DJ, Sabatier P. Mapping Rift Valley Fever vectors and prevalence using rainfall variations. *Vector-Borne Zoonot*. 2004;4(1):33–42. 10.1089/153036604773082979 15018771

[22] Linthicum K, Britch S, Anyamba A, Berenbaum M. Rift Valley Fever: An Emerging Mosquito-Borne Disease. *Annu Rev Entomol*. 2016;61:395 10.1146/annurev-ento-010715-023819 26982443

[23] Murithi RM, Munyua P, Ithondeka PM, Macharia JM, Hightower A, Luman ET, et al Rift Valley fever in Kenya: History of epizootics and identification of vulnerable districts. *Epidemiol Infect*. 2011;139(3):372–80. 10.1017/S0950268810001020 20478084

[24] Tchouassi DP, Bastos AD, Sole CL, Diallo M, Lutomiah J, Mutisya J, et al Population genetics of two key mosquito vectors of rift valley Fever virus reveals new insights into the changing disease outbreak patterns in Kenya. *PLoS Negl Trop Dis*. 2014;8(12):e3364 10.1371/journal.pntd.0003364 25474018 PMC4256213

[25] Rosmoser W, Oviedo M, Lerdthusne E, Patrican L, Turell M, Dohm D, et al Rift Valley fever virus-infected mosquito ova and associated pathology: Possible implications for endemic maintenance. *Res Rep Trop Med*. 2011;2:121–7. 10.2147/RRTM.S13947 30881185 PMC6415639

[26] Linthicum KJ, Davies FG, Bailey CL, Kairo A. Mosquito species encountered in a flooded grassland dambo in Kenya. *Mosq News*. 1984;44(2):228–32.

[27] Lutomiah J, Bast J, Clark J, Richardson J, Yalwala S, Oullo D, et al Abundance, diversity, and distribution of mosquito vectors in selected ecological regions of Kenya: Public health implications. *J Vector Ecol*. 2013;38(1):134–42. 10.1111/j.1948-7134.2013.12019.x 23701618

[28] Linthicum KJ, Bailey CL, Davies FG, Kairo A. Observations on the dispersal and survival of a population of *Aedes-Lineatopennis* (Ludlow) (Diptera, Culicidae) in Kenya. *Bull Entomol Res*. 1985;75(4):661–70.

[29] Logan T, Linthicum K, Thande P, Wagateh J, Nelson G, Roberts C. Egg hatching of *Aedes* mosquitoes during successive floodings in a Rift Valley fever endemic area in Kenya. *J Am Mosquito Contr*. 1991;7(1):109–12. 2045800

[30] Camberlin P, Wairoto JG. Intraseasonal wind anomalies related to wet and dry spells during the ''long'' and ''short'' rainy seasons in Kenya. *Theor Appl Climatol*. 1997;58(1–2):57–69.

[31] Camberlin P, Janicot S, Poccard I. Seasonality and atmospheric dynamics of the teleconnection between African rainfall and tropical sea-surface temperature: Atlantic vs. ENSO. *Int J Climatol*. 2001;21(8):973–1005

[32] Edwards F. Mosquitoes of the Ethiopian region III. London, United Kingdom: British Museum of Natural History; 1941.

[33] Gillies M, DeMeillon B. The *Anopholenes* of Africa south of the Sahara. Johannesburg, South Africa: South African Institute of Medical Research; 1968.

[34] Harbach R. The mosquitoes of the subgenus *Culex* in Southwestern Asia and Egypt (Diptera: Culicidae). *Contr Am Entomol Inst*. 1988;24:240.

[35] Jupp P. Mosquitoes of southern Africa. Hartebeespoort, South Africa: Ecogilde; 1986.

[36] Wan Z, Zhang Y, Zhang Q, Li Z. Validation of the land-surface temperature products retrieved from Terra Moderate Resolution Imaging Spectroradiometer data. *Proc Spie*. 2002;83(1–2):163–80.

[37] Beven K, Kirkby M. A Physically-based Variable Contributing Area Model of Basin Hydrology. *Hydrol Sci Bull*. 1979;24:43–69.

[38] Shangguan W, Dai Y, Duan Q, Liu B, Yuan H. A Global Soil Data Set for Earth System Modeling. *J Adv Model Earth Syst*. 2014;6:249–63.

[39] Hijmans R. raster: Geographic Data Analysis and Modeling. R package version 25–2. 2015; https://CRAN.R-project.org/package=raster.

[40] Ridout M, Demetrio G, Hinde J. Models for Count Data with Many Zeros. Proceedings of XIXth International Biometric Society Conference Cape Town, South Africa International Biometric Society; Wasthington, D.C., USA 1998. p. 179–92.

[41] Zuur AF. Mixed effects models and extensions in ecology with R. New York, NY: Springer; 2009 xxii, 574 p.p.

[42] Lambert D. Zero-Inflated Poisson regression, with an application to defects in manufacturing. *Technometrics*. 1992;34:1–14.

[43] Yeşilova A, Kaydan MB, Kaya Y. Modeling insect-egg data with excess zeros using zero-inflated regression models. *Hacet J Math Stat*. 2010;39:273–82.

[44] Desouhant E, Debouzie D, Menu F. Oviposition pattern of phytophagous insects: On the importance of host population heterogeneity. *Oecologia*. 1998;114(3):382–8. 10.1007/s004420050461 28307782

[45] Martin TG, Wintle BA, Rhodes JR, Kuhnert PM, Field SA, Low-Choy SJ, et al Zero tolerance ecology: Improving ecological inference by modelling the source of zero observations. *Ecol Lett*. 2005;8:1235–46. 10.1111/j.1461-0248.2005.00826.x 21352447

[46] Akaike H. Likelihood of a model and information criteria. *J Econometrics*. 1981;16:3–14.

[47] Burnham KP, Anderson DR, Burnham KP. Model selection and multimodel inference: A practical information-theoretic approach 2nd ed New York: Springer; 2002 xxvi, 488 p.p.

[48] Schwarz G. Estimating Dimension of a Model. *Ann Stat*. 1978;6:461–4.

[49] Zeileis A, Kleiber C, Jackman S. Regression models for count data in R. *J Stat Softw*. 2008;27:1–25.

[50] Bjornstad O. ncf: spatial nonparametric covariance functions. R package version 11–6. 2015; https://CRAN.R-project.org/package=ncf.

[51] Bjornstad ON, Falck W. Nonparametric spatial covariance functions: Estimation and testing. *Environ Ecol Stat*. 2001;8:53–70.

[52] Zuur A, Saveliev A, Ieno E. Zero Inflated Models and Generalized Linear Mixed Models with R. Newburgh, United Kingdom: Highland Statistics, Ltd; 2012.

[53] Shmueli G. To Explain or to Predict? *Stat Sci*. 2010;25:289–310.

[54] Sober E. Instrumentalism, parsimony, and the Akaike framework. *Philos Sci*. 2002;69:S112–S23.

[55] Turell MJ. Effect of environmental temperature on the vector competence of *Aedes fowleri* for Rift Valley fever virus. *Res Virol*. 1989;140:147–54. 10.1016/s0923-2516(89)80092-5 2756242

[56] Turell MJ, Rossi CA, Bailey CL. Effect of extrinsic incubation-temperature on the ability of *Aedes taeniorhynchus* and *Culex pipiens* to transmit Rift-Valley fever virus. *Am J Trop Med Hy*g. 1985;34:1211–8. 10.4269/ajtmh.1985.34.1211 3834803

[57] Anyamba A, Small J, Britch S, Tucker C, Pak E, Reynolds C, Crutchfield J, Linthicum KJ. Recent weather extremes and impacts on agricultural production and vector-borne disease outbreak patterns. *PLoS One*. 2014;9(3). 10.1371/journal.pone.0092538 PMC3962414 24658301

[58] Linthicum K, Anyamba A, Britch S, Small J, Tucker C. Climate Teleconnections, Weather Extremes, and Vector-Borne Disease Outbreaks. Global Health Impacts of Vector-Borne Diseases, Workshop Summary.Forum on Microbial Threats, National Academy of Medicine; 2016. p. 183–200.

[59] Centers for Disease Control and Prevention. Rift Valley fever outbreak—Kenya, November 2006-January 2007. MMWR *Morb Mortal Wkly Rep*. 2007;56:73–6. 17268404

[60] Munyua P, Murithi RM, Wainwright S, Githinji J, Hightower A, Mutonga D, Macharia J, Ithondeka PM, Musaa J, Breiman RF, Bloland P, Njenga MK. Rift Valley fever outbreak in livestock in Kenya, 2006–2007. *Am J Trop Med Hyg*. 2010;83:58–64. 10.4269/ajtmh.2010.09-0292 20682907 PMC2913503

[61] World Health Organization. Outbreaks of Rift Valley fever in Kenya, Somalia and United Republic of Tanzania, December 2006-April 2007. *Wkly Epidemiol Rec*. 2007;82:169–78. 17508438

[62] Nderitu L, Lee JS, Omolo J, Omulo S, O’Guinn ML, Hightower A, Mosha F, Mohamed M, Munyua P, Nganga Z, Hiett K, Seal B, Feikin DR, Breiman RF, Njenga MK. Sequential Rift Valley fever outbreaks in eastern Africa caused by multiple lineages of the virus. *J Infect Dis*. 2011;203:655–65. 10.1093/infdis/jiq004 21282193 PMC3071275

[63] Nguku PM, Sharif SK, Mutonga D, Amwayi S, Omolo J, Mohammed O, Farnon EC, Gould LH, Lederman E, Rao C, Sang R, Schnabel D, Feikin DR, Hightower A, Njenga MK, Breiman RF. An investigation of a major outbreak of Rift Valley fever in Kenya: 2006–2007. *Am J Trop Med Hyg*. 2010;83(2 Suppl):5–13. 10.4269/ajtmh.2010.09-0288 20682900 PMC2913496

[64] Anyamba A, Small J, Tucker C, Linthicum K, Chretien K. Possible RVF activity in the Horn of Africa. Emergency Prevention System for Trans-boundary Animal and Plant Pests and Diseases (EMPRES), Food and Agricultural Organization of the United Nations; 2006.

[65] World Health Organization. Rift Valley fever—Kenya In: News DO, editor. 2018.

[66] ProMed. Rift Valley Fever—Kenya: Alert, Prevention. In: 20180531.5830703, editor. 2018.

[67] ProMed-mail. Rinderpest—Kenya: OIE, suspected. ProMed-mail 2002. 2002;01 Nov: 20021101.5682(<http://www.promedmail.org>).

[68] FAO, OIE, WHO. Africa—El Niño and increased risk of Rift Valley fever—Warning to countries. EMPRES WATCH. 2015;34(December 2015. Rome).

[69] Brown H, Paladini M, Cook R, Kline D, Barnard D, Fish D. Effectiveness of mosquito traps in measuring species abundance and composition. *J Med Ent*. 2008;45:517–21. 10.1603/0022-2585(2008)45[517:eomtim]2.0.co;2 18533447

