# Supplementary figures and images for: Predicting Abundances of Aedes mcintoshi, a primary Rift Valley fever virus mosquito vector
Source: PLoS One. 2019 Dec 17;14(12):e0226617. doi: 10.1371/journal.pone.0226617 (PMC6917266; doi:10.1371/journal.pone.0226617)

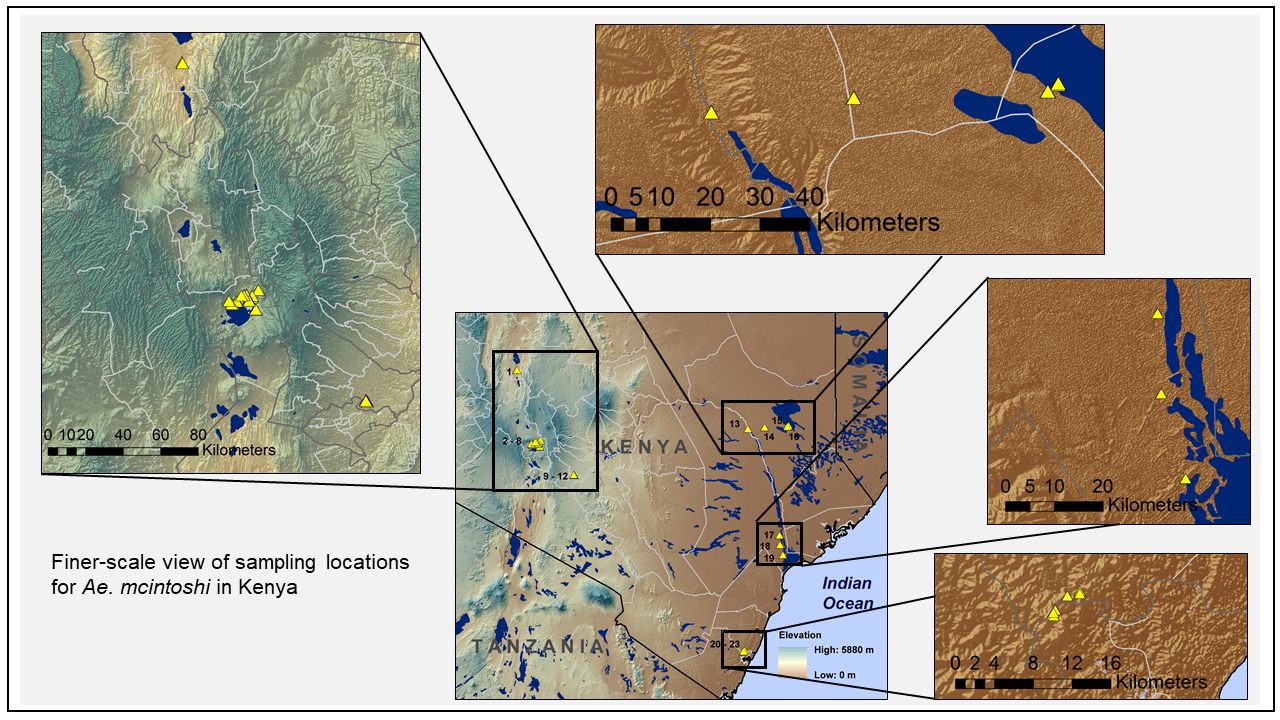

Supplement: S1 Fig — (TIF) [file pone.0226617.s001.tif]

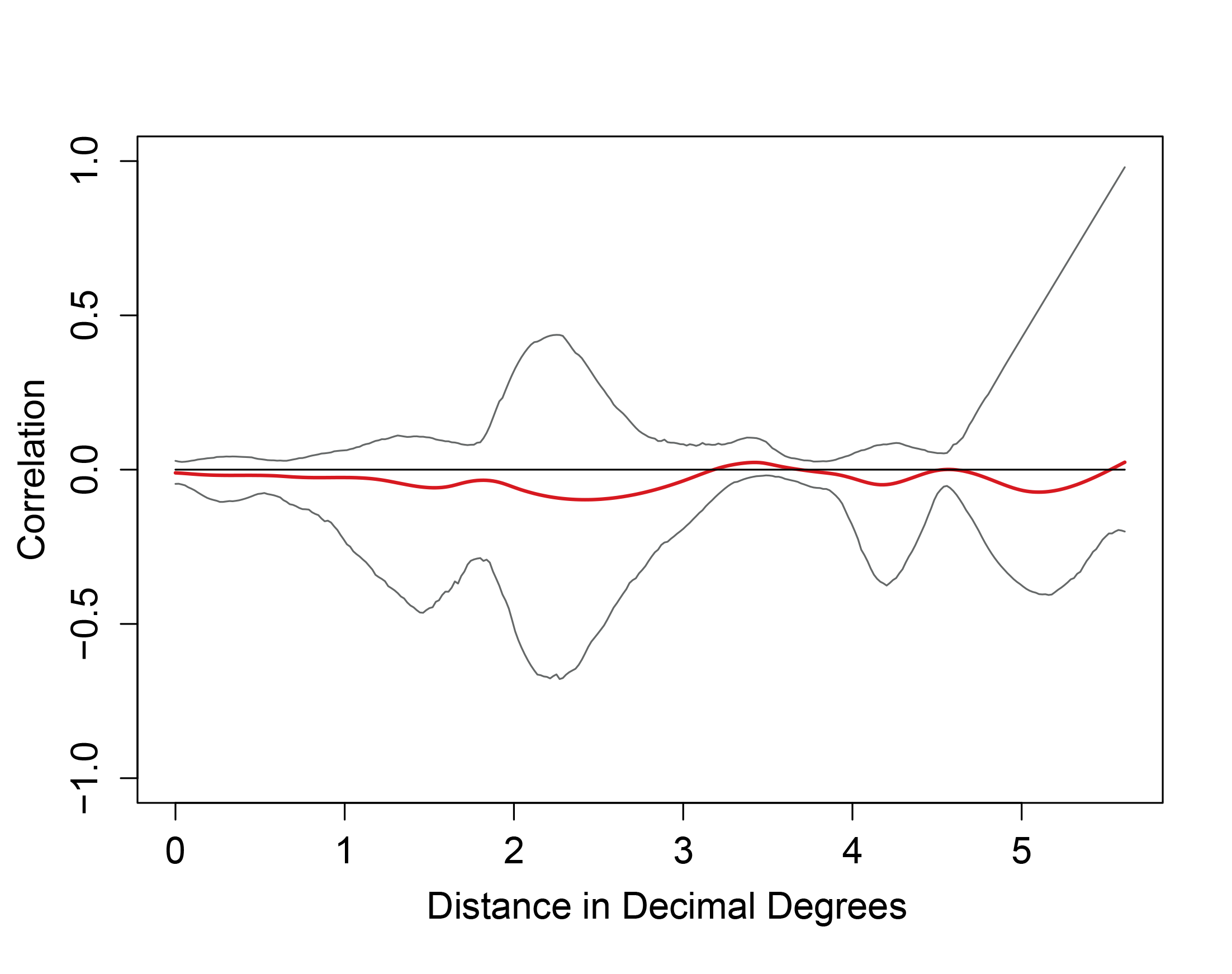

Supplement: S2 Fig — The horizontal black line located at 0.0 on the y-axis represents complete spatial randomness; the red line plots the residual correlation from the model over distance; and the additional black lines show 95% bootstrap confidence intervals around the observed residual correlation. (TIF) [file pone.0226617.s002.tif]
